# Public Opinion on Nutrition-Related Policies to Combat Child Obesity, Los Angeles County, 2011

**DOI:** 10.5888/pcd11.140005

**Published:** 2014-06-05

**Authors:** Paul A. Simon, Choiyuk Chiang, Amy S. Lightstone, Margaret Shih

**Affiliations:** Author Affiliations: Choiyuk Chiang, Amy S. Lightstone, Margaret Shih, Los Angeles County Department of Public Health, Los Angeles, California.

## Abstract

We assessed public opinion on nutrition-related policies to address child obesity: a soda tax, restrictions on advertising unhealthy foods and beverages to children, and restrictions on siting fast food restaurants and convenience stores near schools. We analyzed data from 998 adults (aged ≥18 years) in the 2011 Los Angeles County Health Survey. Support was highest for advertising restrictions (74%), intermediate for a soda tax (60%), and lowest for siting restrictions on fast food restaurants and convenience stores (44% and 37%, respectively). Support for food and beverage advertising restrictions and soda taxation is promising for future policy efforts to address child obesity.

## Objective

Efforts to reverse the obesity epidemic are unlikely to be successful without policy interventions that address “toxic” food environments (1). Although most nutrition policies to address the child obesity epidemic have focused on school, preschool, and childcare settings, national guidelines also include policy strategies in community settings (2). Only limited information is available on the comparative levels of support among these policy options (3). To address this limitation, we examined public opinion in Los Angeles County on 4 prominent nutrition-related policy strategies in community settings to address child obesity: sugar-sweetened beverage taxation, restrictions on unhealthy food and beverage marketing to children, restrictions on fast food restaurants near schools, and restrictions on convenience stores near schools.

## Methods

We analyzed data from the 2011 Los Angeles County Health Survey (LACHS), a random-digit–dialed telephone (landline and cellular) survey of the noninstitutionalized county population (4). The survey was approved by the Los Angeles County Department of Public Health’s Institutional Review Board.

Among all eligible households contacted for the survey, interviews were completed with 66% (n = 8,036). The present study included a randomly selected subsample of 998 adults (aged ≥18 years) who were asked whether they agreed or disagreed with the following 4 statements: 1) I would support a tax increase on sodas as a way to discourage kids and others from drinking too many of them; 2) There should be restrictions placed on the advertising of sugared cereals, candy, soda, and fast food to children; 3) There should be a law prohibiting fast food restaurants within a quarter-mile of schools; and 4) There should be a law prohibiting convenience stores within a quarter-mile of schools. Interviews were conducted in English, Spanish, Cantonese, Mandarin, Vietnamese, and Korean.

The data were weighted to reduce potential bias associated with differential selection and response (4). The percentage of adults who agreed with each policy statement was calculated for the total study group and by sex, age group, race/ethnicity, education level, and annual household income relative to the federal poverty level. Ninety-five percent confidence intervals were calculated for each point estimate. All analyses were conducted by using SAS (version 9.2, SAS Institute Inc, Cary, North Carolina).

## Results

Overall, restrictions on unhealthy food and beverage advertising received the highest level of support (74%), followed by a soda tax (60%) (Table). Restrictions on siting fast food restaurants and convenience stores near schools had considerably less support (44% and 37%, respectively). Women were more supportive of the policies than men. Among Latinos, support was greater among those from Spanish-speaking households than English-speaking households for all 4 policy statements.

**Table:** Respondents Who Agreed with Nutrition Policy Statements by Sociodemographic Characteristic, Los Angeles County Health Survey, 2011

Characteristic	Soda Tax		Restrict Food Marketing		Prohibit Fast Food Near Schools		Prohibit Convenience Stores Near Schools	
	n^a^	% Agree (95% CI)	n^a^	% Agree (95% CI)	n^a^	% Agree (95% CI)	n^a^	% Agree (95% CI)
Overall	972	60.1 (55.3–64.8)	973	74.2 (70.0–78.4)	957	43.5 (38.5–48.5)	956	37.0 (32.2–41.9)
**Sex**								
Male	371	57.0 (49.5–64.6)	370	68.2 (61.0–75.3)	366	37.2 (29.7–44.8)	368	34.8 (27.3–42.3)
Female	601	62.9 (57.1–68.8)	603	79.7 (75.1–84.4)	591	49.3 (42.8–55.7)	588	39.2 (32.8–45.5)
**Age group, y**								
18–29	121	61.1 (49.6–72.6)	123	69.5 (58.3–80.8)	121	45.4 (33.4–57.5)	123	24.5 (14.9–34.1)
30–49	358	63.4 (55.9–70.9)	358	78.5 (72.7–84.3)	355	43.6 (35.4–51.7)	356	42.2 (33.9–50.5)
50–64	268	57.1 (48.6–65.7)	267	70.7 (62.9–78.5)	265	42.3 (33.4–51.2)	261	39.4 (30.7–48.1)
≥65	225	53.4 (43.2–63.6)	225	75.8 (67.1–84.5)	216	42.0 (31.8–52.2)	216	40.8 (30.6–51.1)
**Race/Ethnicity**								
Latino	327	68.1 (60.6–75.6)	325	76.0 (69.2–82.8)	325	53.5 (45.1–61.8)	319	38.3 (30.5–46.0)
White	431	46.5 (39.0–54.0)	429	74.6 (68.9–80.3)	424	30.9 (24.3–37.4)	427	26.8 (19.6–34.1)
African American	113	50.2 (36.8–63.5)	113	72.3 (59.5–85.1)	109	44.7 (31.2–58.1)	108	41.1 (27.7–54.5)
Asian/Pacific Islander	91	71.6 (59.7–83.4)	96	70.2 (56.8–83.5)	89	41.3 (27.2–55.5)	92	53.7 (39.4–68.1)
**Language of interview among Latinos**								
English-speaking	178	52.9 (41.0–64.8)	178	67.6 (56.5–78.7)	177	40.8 (29.5–52.2)	179	28.1 (18.9–37.2)
Spanish-speaking	149	79.5 (70.6–88.4)	147	82.4 (74.2–90.7)	148	62.8 (51.7–73.9)	140	46.5 (35.0–58.0)
**Education**								
Less than high school graduate	163	72.6 (63.2–82.0)	164	81.7 (73.5–89.9)	161	56.0 (44 6–67.5)	157	39.9 (28.8–50.9)
High school graduate	151	66.5 (56.2–76.8)	152	70.5 (59.7–81.3)	144	49.7 (37.5–61.9)	147	42.9 (31.0–54.9)
Some college or trade school	272	46.8 (38.3–55.3)	272	71.8 (63.9–79.6)	268	40.2 (31.7–48.7)	269	33.2 (25.2–41.2)
College or postgraduate degree	379	58.1 (49.8–66.4)	379	73.1 (66.1–80.1)	377	30.7 (23.4–38.1)	377	33.7 (24.9–42.6)
**Income^b^**								
0%–99% FPL	161	69.2 (58.7–79.7)	162	69.9 (59.3–80.4)	156	58.5 (46.9–70.2)	157	45.0 (33.4–56.6)
100%–199% FPL	193	73.7 (64.9–82.6)	192	81.9 (73.5–90.4)	187	48.0 (36.7–59.3)	187	43.4 (32.1–54.6)
200%–299% FPL	126	48.3 (35.5–61.0)	127	71.6 (59.2–84.0)	126	45.9 (33.1–58.7)	124	34.6 (22.3–46.9)
300% or above FPL	492	49.3 (42.5–56.1)	492	72.0 (66.4–77.6)	488	31.7 (25.5–37.9)	488	29.3 (23.3–35.3)

Abbreviation: CI, confidence interval.

a Counts may not sum to overall totals because of missing data.

b Income was categorized based on US Census 2009 federal poverty level (FPL) thresholds, which for a family of 4 (2 adults, 2 dependents) correspond to annual incomes of $21,756 (100% FPL), $43,512 (200% FPL), and $65,268 (300% FPL).

Variation in support was also observed across other demographic subgroups but differed by policy statement. For the soda tax, support was greater among Latinos and Asians/Pacific Islanders than among whites and African Americans and was higher among those with less education and lower household incomes. For advertising restrictions, support was high across all demographic subgroups. For restrictions on fast food restaurants and convenience stores near schools, support was lowest among whites and those with higher household incomes. Support for restrictions on convenience stores was lower among adults aged 18 to 29 years than among older adults.

## Discussion

Our findings suggest that efforts to restrict unhealthy food and beverage marketing to children may have broad public support if focused specifically on advertising restrictions. There is strong evidence that this marketing has a significant adverse impact on dietary patterns in children and may be an important contributor to the obesity epidemic (5). Although there are legal considerations in pursuing public policy that would regulate food and beverage marketing to children (6), our results suggest that such efforts would receive support. This support may help persuade private companies to self-regulate (7). There may also be opportunities to expand advertising restrictions in public schools and publicly regulated child care settings. For example, the White House and US Department of Agriculture recently announced proposed federal restrictions on unhealthy food and beverage advertising in public schools (8).

We also found that nearly two-thirds of adults in the county support taxation of soda as a means of reducing its consumption. However, the defeats by wide margins of 2 sugar-sweetened beverage tax initiatives in California in 2012 (9), including 1 in the City of El Monte in Los Angeles County, suggest that this support may not be sustained in the face of strong opposition, highlighting the need for robust and well-coordinated advocacy efforts. In addition, our results suggest that these efforts are unlikely to be successful unless they are able to generate greater support among whites, African Americans, and those in middle-income and more affluent households.

The low public support for restrictions on siting fast food restaurants and convenience stores near schools suggests that using zoning and other land use regulation to improve food environments may be challenging. Because this strategy is focused primarily on new establishments, its impact may be relatively limited in urban centers where large numbers of pre-existing fast food restaurants and convenience stores are near schools.

Our study has several limitations. First, although Los Angeles County comprises an ethnically and socioeconomically diverse urban, suburban, and rural population, the results may not be generalizable to other jurisdictions. For example, a national online survey of parents found only modest support for food advertising restrictions, although the measures used were different from our survey and the results, therefore, are not directly comparable (10). In addition, our findings on food advertising restrictions and sugar-sweetened beverage taxation are consistent with those of several statewide surveys in California (11,12), suggesting that our results may reflect more widespread views. Second, because the survey collected only information on whether respondents agreed or disagreed with the policy statements, we cannot assess the intensity of support or opposition to these statements. Despite this limitation, our results indicate the levels of support across the 4 policy areas, providing direction for prioritizing policy efforts.
